# Prostate Artery Embolization for Benign Prostatic Hyperplasia: A Case Report and Comprehensive Literature Review

**DOI:** 10.7759/cureus.77182

**Published:** 2025-01-09

**Authors:** Om Prakash Bhatta, Rutva Jani, Akhil Dhanjibhai Kakadiya, Iram Fatima, Shubh Mehta, Alen Sam

**Affiliations:** 1 Department of Anaesthesiology, Tribhuvan University Institute of Medicine, Kathmandu, NPL; 2 Department of Internal Medicine, C. U. Shah Medical College and Hospital, Gujarat, IND; 3 Department of Internal Medicine, Gujarat Medical Education and Research Society (GMERS) Medical College &amp; Hospital, Sola, Ahmedabad, IND; 4 Department of Internal Medicine, Holy Name Medical Center, Teaneck, USA; 5 Department of Internal Medicine, B. J. Medical College and Civil Hospital, Ahmedabad, IND; 6 Department of Internal Medicine, Government Medical College, Kozhikode, Kozhikode, IND

**Keywords:** and bladder (kub), benign prostatic hyperplasia (bph), kidneys, lower urinary tract symptoms (luts), minimally invasive surgical technique (mist), prostate artery embolization (pae), ureters

## Abstract

This case report describes the successful use of prostate artery embolization (PAE) in a 75-year-old male with benign prostatic hyperplasia (BPH) and severe lower urinary tract symptoms (LUTS) that did not respond to medical therapy. Despite initial treatments, the patient had persistent symptoms and significant prostate enlargement. PAE was chosen for its minimally invasive nature and advantages over traditional surgery. After the procedure, the patient experienced significant improvements, including reduced prostate volume, decreased post-void residual volume, and enhanced urinary flow. Follow-up showed a marked reduction in symptoms. This case highlights PAE as an effective alternative for patients with BPH who do not respond to medical management or are not suitable for conventional surgery. Further research is needed to evaluate long-term outcomes compared to established treatments.

## Introduction

Benign prostatic hyperplasia (BPH) is characterized by non-malignant enlargement of the transitional zone of the prostate gland, which manifests as lower urinary tract symptoms (LUTS). BPH is a common condition among aging men, with 50% of men over 50 years of age showing evidence of BPH [1]. Alpha-blockers and 5-alpha reductase inhibitors are two medication treatments for BPH, in addition to surgical methods such as transurethral resection of the prostate (TURP). Although these surgical methods work well, there is a risk of bleeding, infection, and sexual dysfunction with these procedures, which has led to researchers looking for less intrusive options [2]. 

Prostate artery embolization (PAE) has emerged as a minimally invasive treatment option for benign prostatic hyperplasia (BPH), offering a suitable alternative for patients who are not candidates for surgery. PAE works by selectively blocking the blood vessels that supply the prostate, leading to a decrease in prostate size and an improvement in urinary symptoms. Research has consistently demonstrated that PAE is a safe and effective procedure, associated with fewer complications than traditional surgical methods, while providing notable symptom relief and enhancing patient’s quality of life [3,4]. The procedure results in a significant reduction in mean prostate volume and is associated with a low complication rate, with major complications occurring in less than 5% of patients [3]. However, it remains a relatively novel treatment, with long-term outcomes and patient selection criteria being areas of active research. 

In this report, we present the case of a 75-year-old male with BPH who presented with moderate to severe LUTS was unresponsive to medical therapy, and underwent PAE using Embospheres 300-500 um, resulting in significant symptomatic relief and no major complications.

## Case presentation

We report the case of a 75-year-old male who presented to the urology outpatient clinic with pronounced lower urinary tract symptoms (LUTS), including incomplete bladder emptying, a weak urinary stream, and frequent urination. Despite receiving prior medical therapy, his symptoms persisted, significantly impacting his quality of life. His International Prostate Symptom Score (IPSS) was 22, indicating moderate to severe LUTS. On physical examination, the prostate was found to be enlarged and firm, without any nodules or tenderness. Initial investigations included laboratory tests and diagnostic imaging, as shown in Table 1.

**Table 1:** Laboratory parameters of the patient. PT: prothrombin time, INR: international normalized ratio.

Test	Result	Normal range
Prostate-specific antigen (PSA)	3.05 ng/ml	<4.0 ng/ml
Creatinine	1.1 mg/dl	0.7-1.3 mg/dl
Urea	40 mg/dl	7-20 mg/dl
INR	1.09	0.8-1.2
PT	13.1 sec	11-14 sec
Total leukocyte count (TLC)	9.08 K/µl	4.0-10.0 K/µl
Hemoglobin (Hb)	11 g/dl	13.8-17.2 g/dl (Male)

**Figure 1:**
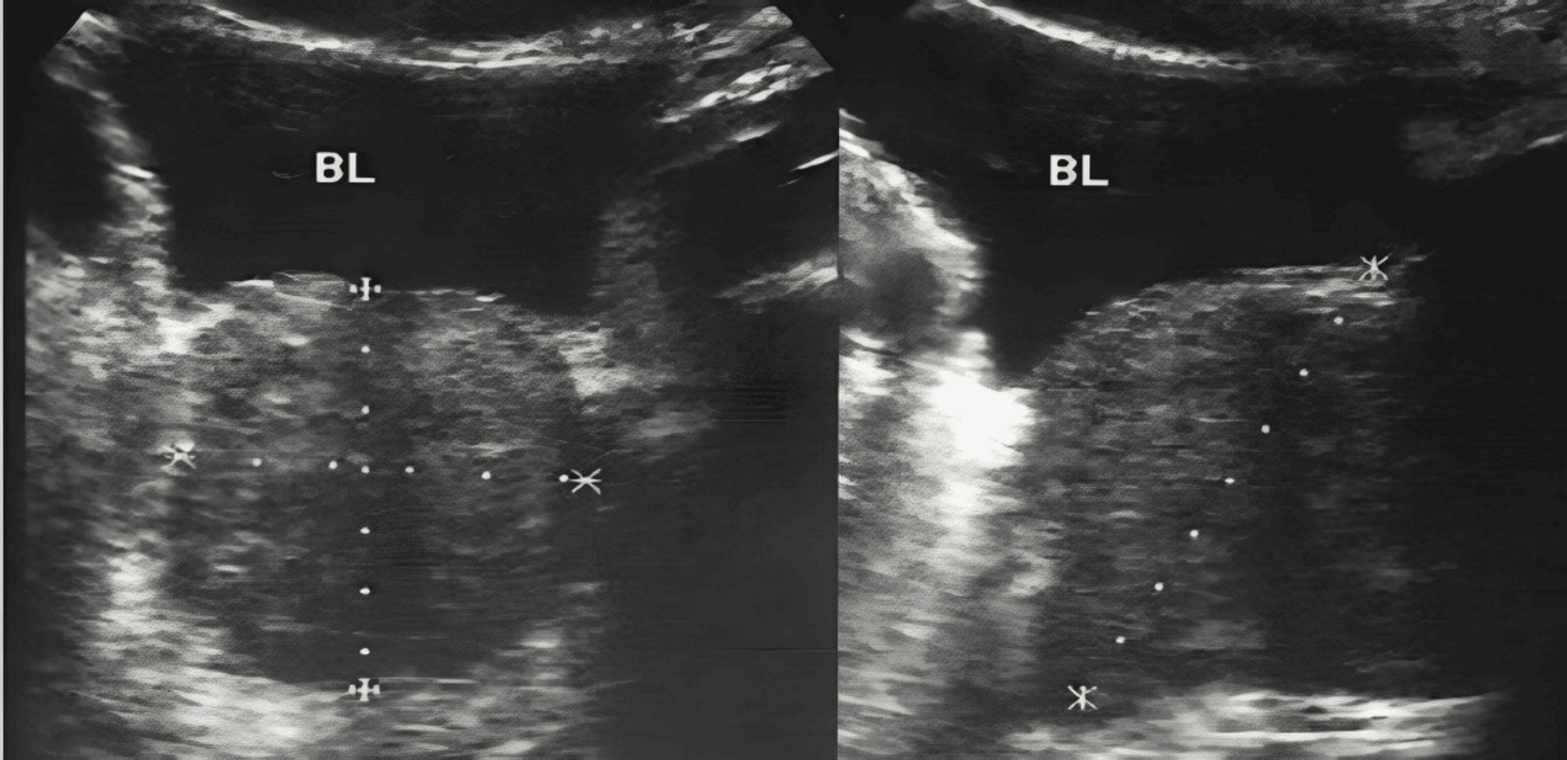
Ultrasound of the kidneys, ureters, and bladder (USG KUB) showing Grade III prostatomegaly with a homogeneous echotexture. BL: bladder.

Uroflowmetry showed a peak flow rate of 7 ml/s. An ultrasound of the kidneys, ureters, and bladder (KUB) revealed Grade III prostatomegaly with a prostate volume of 62 cc and significant post-void residual urine volume (PVRV) of 180 cc, as shown in Figure 1.

**Figure 2:**
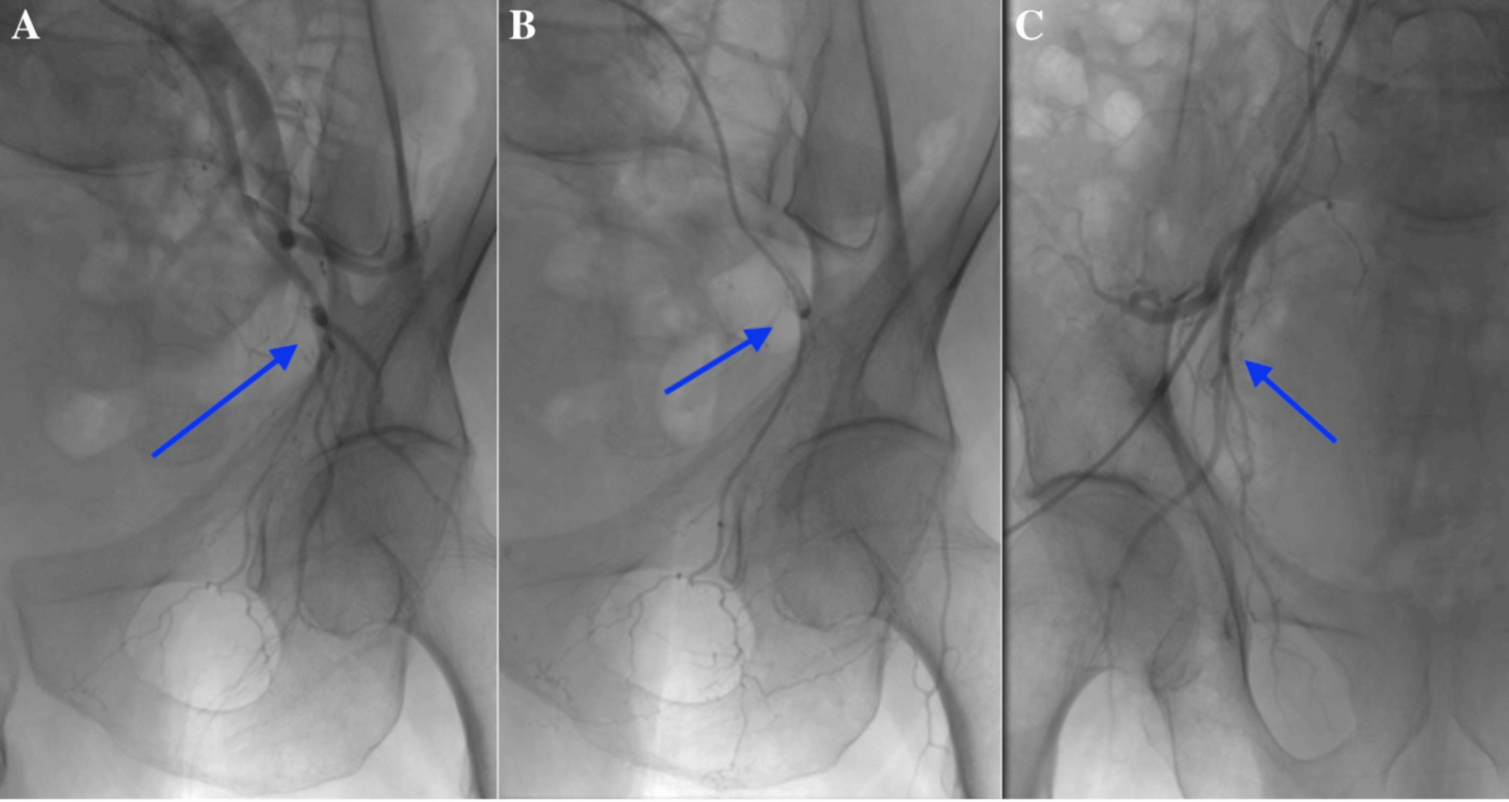
Angiographic assessment of the iliac vessels and prostate arteries. (A) Pelvic angiography showing the iliac vessels. (B) Angiographic image of the left internal iliac artery, revealing the left inferior vesical artery (prostate artery) originating from the anterior division of the left internal iliac artery, consistent with the type II variant. (C) Angiographic image of the right internal iliac artery, demonstrating the right inferior vesical artery arising from the right internal pudendal artery, corresponding to the type IV variant. These images (blue arrows) highlight the anatomical variations in the vascular supply to the prostate between the two sides of the pelvis.

Given the patient's resistance to medical management, prostate artery embolization (PAE) was selected as a minimally invasive treatment option. The pre-procedure evaluation confirmed his eligibility for PAE. The procedure began with a CT pelvic angiography to assess the iliac vessels (Figure 2A). Under local anesthesia, a femoral artery access was established using a 5F arterial sheath (Terumo Corporation, Tokyo, Japan). The angiographic examination revealed distinct anatomical variations in the arteries supplying the prostate. Specifically, Figure 2B shows that the left inferior vesical artery (which supplies the prostate) originated from the anterior division of the left internal iliac artery, consistent with the type II variant. Conversely, Figure 2C illustrates that the right inferior vesical artery arose from the right internal pudendal artery, representing the type IV variant. These findings underscore the variations in vascular supply to the prostate between the two sides of the pelvis.

**Figure 3:**
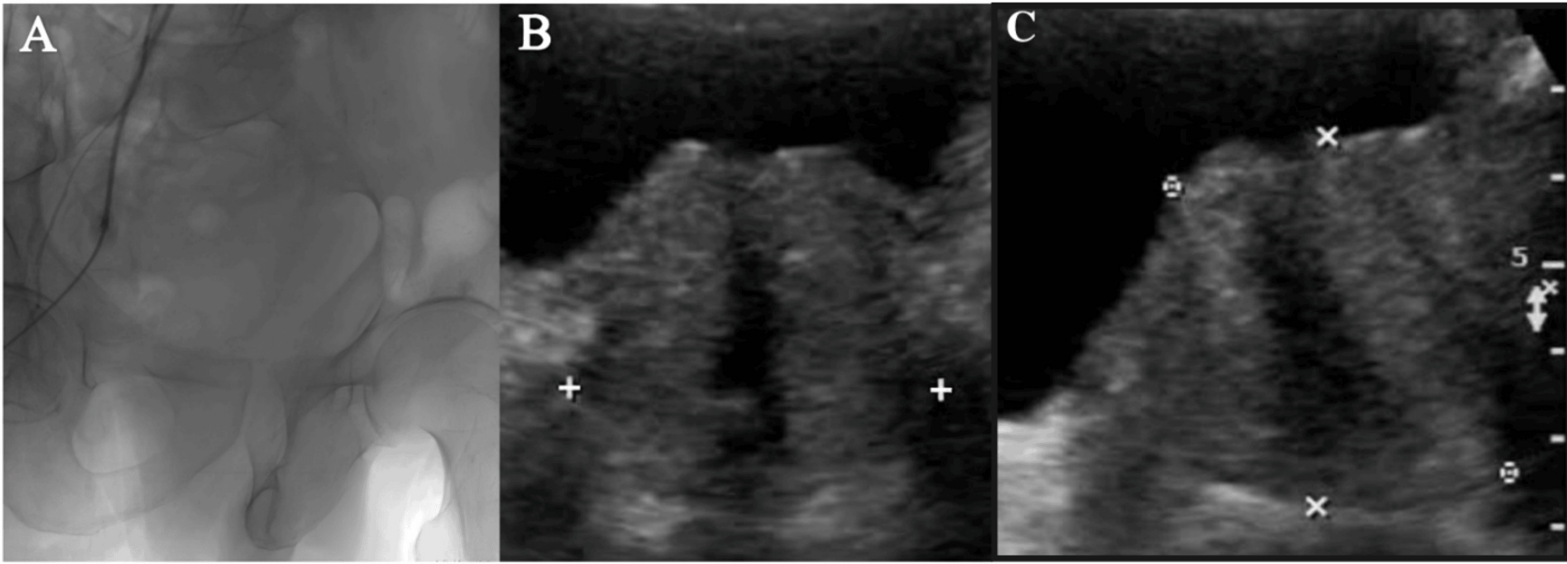
Embolization and post-procedure imaging. (A) Embospheres are used to embolize the inferior vesical artery and its intraprostatic branches. (B) Ultrasound of the kidneys, ureters, and bladder (USG KUB) showing the prostate with a volume of 42 cc, homogenous echotexture, and a significantly decreased post-void residual volume (PVRV) of 40 ml.

A microcatheter was advanced into this artery, and a vasodilator was used to facilitate navigation. Embospheres were injected in high dilution, with the microcatheter positioned distally in the prostatic branches for embolization. The procedure was repeated on the right side, where the right inferior vesical artery was found to arise from the right internal pudendal artery (type IV variant) (Figure 3A).

The PAE procedure was uneventful, and the patient was moved to the ward with stable vitals. After a three-month follow-up, the patient reported significant improvement in LUTS. His International Prostate Symptom Score (IPSS) score decreased to eight, indicating mild symptoms. Follow-up imaging showed a reduction in prostate volume to 42 cc, a decrease in PVRV to 40 ml, and an improvement in peak flow rate to 13 ml/s (Figure 3B). The patient did not experience any adverse effects from the procedure.

## Discussion

This case illustrates the successful use of PAE in treating a 75-year-old male with BPH who presented with moderate to severe LUTS unresponsive to medical therapy. BPH is characterized by non-malignant enlargement of the transitional zone of the prostate gland, leading to bladder outlet obstruction, which manifests as LUTS, including a weak urinary stream, frequent urination, and incomplete bladder emptying. BPH is a common condition among aging men, with 50% of men over 50 showing evidence of BPH [1,3]. LUTS and bladder outlet obstruction in men with BPH arise from static and dynamic factors. The International Prostatic Symptom Score (IPSS) is universally accepted for quantifying the quality of life and severity of symptoms [5].

The treatment approach for BPH depends on the severity of symptoms and individual patient factors. In cases of mild symptoms, a strategy of watchful waiting is often advised, which includes lifestyle modifications such as reducing fluid intake, performing pelvic floor exercises, and addressing underlying contributing factors. For patients with more significant symptoms, medical therapy is the mainstay. Alpha-blockers are commonly prescribed to relax the prostatic smooth muscles, improving urine flow and reducing LUTS. In cases where the prostate is larger than 30 g, 5-alpha reductase inhibitors are effective, though they require a longer period to achieve full efficacy. These inhibitors can be used in combination with alpha-blockers to enhance symptom relief compared to monotherapy. Additional treatments include phosphodiesterase inhibitors, antimuscarinics, and beta-3 agonists as complementary options [6].

Surgery is a key option for patients who do not respond to or cannot tolerate medical therapy, those with severe disease, or those who choose to avoid medical treatment. Standard guidelines recommend surgery for patients with upper urinary tract complications (e.g., hydronephrosis and renal insufficiency), refractory urinary retention, recurrent urinary tract infections, bladder decompensation, recurrent bladder calculi, or persistent gross hematuria requiring surgical intervention. The choice of procedure should be based on the size and shape of the prostate gland, the patient's bleeding risk, presentation (e.g., concurrent stones, symptom severity), and the patient’s attitude toward potential sexual side effects. Transurethral resection of the prostate (TURP) remains the gold standard surgical treatment for BPH, effectively relieving symptoms by resecting obstructing prostatic tissue. Recent advances have introduced minimally invasive alternatives such as PAE, laser vaporization, and holmium laser enucleation of the prostate (HoLEP), which offer similar efficacy with reduced perioperative risks, particularly in larger prostates [6,7].

PAE is a minimally invasive surgical technique (MIST) for BPH and has emerged as a promising alternative to traditional surgical treatments in patients with moderate to severe LUTS, especially for those who are poor surgical candidates or seek less invasive options. Studies have shown that PAE is a safe procedure that reduces prostate volume, improves clinical symptoms, and enhances the quality of life of patients, with low rates of complications [1,7,8].

In the current case, the patient's peak flow rate improved from 7 ml/s to 13 ml/s post-procedure, consistent with findings from other studies that have documented similar improvements in uroflowmetry following PAE. Our patient also reported improvements in symptoms, quality of life, and sexual function at the most recent follow-up and marked improvement in the IPSS score.

The pre-procedure evaluation for PAE in BPH patients involves excluding other causes of bladder outflow obstruction and LUTS, such as prostate cancer, neurologic abnormalities, and urinary tract infections. It includes physical exams, questionnaires, lab tests, and imaging like ultrasound (US), computed tomography (CT), and magnetic resonance imaging (MRI). US assesses prostate size, morphology, and post-void residual volume (PVR), while elastography evaluates prostatic elasticity. CT and CTA help visualize the prostatic artery and detect anastomoses, though MRI is preferred for detailed anatomical assessment and identifying potential alternate causes of symptoms. These evaluations ensure accurate diagnosis and effective PAE planning [6]. Table 2 below summarizes the key characteristics, reasons for PAE, patient outcomes, and complications observed in various studies.

**Table 2:** Summary of key studies on prostatic artery embolization (PAE) for BPH with persistent hematuria and LUTS. BPH: benign prostatic hyperplasia, LUTS: lower urinary tract symptoms.

Study	Age	Comorbidities	Reason for PAE	Outcomes	Complications	Follow-up
DeMeritt et al. [9]	63-78 years	Hypertension, diabetes	Hematuria, LUTS	Reduced prostate volume, symptom relief	Mild, self-limited effects	12 months
Carnevale et al. [5]	60-75 years	Diabetes, coronary artery disease	LUTS due to BPH	Symptom relief, improved quality of life	No major complications	Six months
Salem et al. [4]	65-80 years	Hypertension, BPH, obesity	LUTS, Hematuria, BPH	Symptom improvement, increased urinary flow	Minimal, self-limited effects	12 months

Precise identification of the prostatic arteries minimizes the risk of nontarget embolization, a complication that can lead to adverse outcomes. Recent literature has emphasized the importance of careful pre-procedural imaging. Intraprocedural cone beam CT (CBCT) is essential for confirming catheter placement and excluding nontarget embolization, providing critical information not visible during digital subtraction angiography (DSA) in over 60% of cases. Additionally, CBCT has been shown to identify the prostatic artery more accurately and with less radiation exposure compared to conventional pre-procedural CTA [6,10,11].

PAE selectively embolizes the prostatic arteries, causing ischemic necrosis and reducing prostate volume, theoretically lowering blood levels of free testosterone, which may act on prostatic cells. PAE may also reduce alpha-1 adrenergic receptors, resulting in decreased neuromuscular tone [6]. The Proximal Embolization First, Then Embolize Distal (PErFecTED) Technique produces greater prostate ischemia and infarction than classical methods, as well as better clinical improvement of LUTS and lower recurrence rates [11]. The use of embolic agents, such as polyvinyl alcohol (PVA) particles or microspheres of various sizes, has been well documented in the literature, with studies showing comparable efficacy. However, the optimal embolic agent for PAE has yet to be determined [6,11].

Post-procedure evaluation after PAE uses US elastography and MRI to assess treatment success. US elastography measures prostate stiffness and volume reductions, reflecting effective ischemic necrosis of the transition zone tissue. MRI further evaluates prostate volume reduction, changes in signal intensity, and potential complications like nontarget embolization, confirming the effectiveness of PAE [6].

Intraoperative complications include drug or contrast reactions, vascular access issues, and technical failures. Common postoperative complications of PAE include acute urinary retention, dysuria, hematuria, nausea, and vomiting, collectively referred to as 'post-PAE syndrome.' Although rare, serious complications include infection (e.g., prostatitis or abscess), nontarget embolization leading to ischemic injury in the bladder, rectum, or penile arteries, and potential adverse events from radiation, contrast toxicity, and superinfection. Most adverse effects are mild and manageable, with PAE remaining a safe procedure when performed with proper technique and thorough pre-procedural planning [5,11].

The patient experienced no significant complications post-embolization, and there were no reports of common side effects such as transient urinary retention, pelvic pain, or infection. This aligns with the current findings, suggesting that PAE is a safe and well-tolerated procedure with a low complication rate. A meta-analysis by Altman et al. found that PAE has a lower risk of complications than TURP, with lower incidences of sexual dysfunction, urinary incontinence, and other serious adverse events, making it a viable option for patients who are not candidates for surgery [12].

Pisco et al. reported only two serious problems, including bladder ischemia, while Carnevale et al. described a similar condition [5,13]. We experienced no negative effects commonly linked with standard surgical procedures, such as sexual dysfunction or urinary incontinence. TURP has well-documented complication rates ranging from 5 to 15%, but PAE studies have found a lack of problems frequently linked with transurethral therapy, such as bleeding, sexual dysfunction, incontinence, and dilutional hyponatremia [1,4].

This case demonstrates that PAE may be a realistic option for individuals who have not responded to medicinal therapy, are ineligible for surgery or TURP due to other health issues, or have decided against surgery. Although PAE is becoming known as a successful therapeutic option, additional research is required. Long-term effects and comparisons to established treatments, such as TURP and laser therapy, are still being evaluated.

## Conclusions

In conclusion, benign prostatic hyperplasia (BPH) remains a prevalent health issue affecting many men’s quality of life. Prostate artery embolization (PAE) has emerged as a highly effective minimally invasive treatment for BPH-related LUTS that are resistant to medical management. As PAE becomes more widely available, further advancements are anticipated, including refined patient selection criteria, comparative randomized trials, and validation of outcome biomarkers, which will likely enhance its acceptance within the urological community.
